# Impact of the Introduction of a Package of Diagnostic Tools, Diagnostic Algorithm, and Training and Communication on Outpatient Acute Fever Case Management at 3 Diverse Sites in Uganda: Results of a Randomized Controlled Trial

**DOI:** 10.1093/cid/ciad341

**Published:** 2023-07-25

**Authors:** James Kapisi, Asadu Sserwanga, Freddy Eric Kitutu, Elizeus Rutebemberwa, Phyllis Awor, Stephan Weber, Thomas Keller, David Kaawa-Mafigiri, Deborah Ekusai-Sebatta, Philip Horgan, Sabine Dittrich, Catrin E Moore, Olawale Salami, Piero Olliaro, Juvenal Nkeramahame, Heidi Hopkins

**Affiliations:** Department of Disease Surveillance, Infectious Diseases Research Collaboration, Kampala, Uganda; Department of Disease Surveillance, Infectious Diseases Research Collaboration, Kampala, Uganda; Department of Pharmacy, Makerere University School of Health Sciences, Kampala, Uganda; Department of Health Policy, Planning, and Management, Makerere University School of Public Health, Kampala, Uganda; Department of Health Policy, Planning, and Management, Makerere University School of Public Health, Kampala, Uganda; Department of Statistics, ACOMED Statistics, Leipzig, Germany; Department of Statistics, ACOMED Statistics, Leipzig, Germany; Social Work and Social Administration, Makerere University, Kampala, Uganda; Department of Disease Surveillance, Infectious Diseases Research Collaboration, Kampala, Uganda; FIND, Geneva, Switzerland; Nuffield Department of Medicine, Big Data Institute, University of Oxford, Oxford, United Kingdom; Evidence & Impact Oxford, Oxford, United Kingdom; FIND, Geneva, Switzerland; Nuffield Department of Medicine, Centre for Tropical Medicine and Global Health, University of Oxford, Oxford, United Kingdom; Deggendorf Institute of Technology, European-Campus-Rottal-Inn, Pfarrkirchen, Germany; Nuffield Department of Medicine, Big Data Institute, University of Oxford, Oxford, United Kingdom; Centre for Neonatal and Paediatric Infection, Institute for Infection and Immunity, St George's University of London, London, United Kingdom; FIND, Geneva, Switzerland; FIND, Geneva, Switzerland; Nuffield Department of Medicine, Pandemic Sciences Institute, University of Oxford, Oxford, United Kingdom; FIND, Geneva, Switzerland; Department of Disease Control, Faculty of Infectious and Tropical Diseases, London School of Hygiene and Tropical Medicine, London, United Kingdom

**Keywords:** antimicrobial stewardship, antimicrobial resistance, acute febrile illness, point-of-care tests, behavior change

## Abstract

**Background:**

Increasing trends of antimicrobial resistance are observed around the world, driven in part by excessive use of antimicrobials. Limited access to diagnostics, particularly in low- and middle-income countries, contributes to diagnostic uncertainty, which may promote unnecessary antibiotic use. We investigated whether introducing a package of diagnostic tools, clinical algorithm, and training-and-communication messages could safely reduce antibiotic prescribing compared with current standard-of-care for febrile patients presenting to outpatient clinics in Uganda.

**Methods:**

This was an open-label, multicenter, 2-arm randomized controlled trial conducted at 3 public health facilities (Aduku, Nagongera, and Kihihi health center IVs) comparing the proportions of antibiotic prescriptions and clinical outcomes for febrile outpatients aged ≥1 year. The intervention arm included a package of point-of-care tests, a diagnostic and treatment algorithm, and training-and-communication messages. Standard-of-care was provided to patients in the control arm.

**Results:**

A total of 2400 patients were enrolled, with 49.5% in the intervention arm. Overall, there was no significant difference in antibiotic prescriptions between the study arms (relative risk [RR]: 1.03; 95% CI: .96–1.11). In the intervention arm, patients with positive malaria test results (313/500 [62.6%] vs 170/473 [35.9%]) had a higher RR of being prescribed antibiotics (1.74; 1.52–2.00), while those with negative malaria results (348/688 [50.6%] vs 376/508 [74.0%]) had a lower RR (.68; .63–.75). There was no significant difference in clinical outcomes.

**Conclusions:**

This study found that a diagnostic intervention for management of febrile outpatients did not achieve the desired impact on antibiotic prescribing at 3 diverse and representative health facility sites in Uganda.

## Teaser

We report the main findings of the AMR Diagnostics Use Accelerator trial conducted in Uganda to evaluate the impact of a package of interventions on antibiotic prescribing and clinical outcomes for child and adult outpatients with acute febrile illness.

The global burden of antimicrobial resistance (AMR) continues to increase with a growing global population and greater use of and reliance on antimicrobials [1, 2]. A recent report from the Global Research on Antimicrobial Resistance Project estimated that up to 4.95 million deaths globally in 2019 were associated with bacterial AMR. Of these, 1.27 million deaths, more than human immunodeficiency virus (HIV)/AIDS and malaria combined, were directly attributable to resistance using modeled estimates [3]. Increasing trends of AMR are reported in Uganda in the 2015 situation analysis by the Uganda Academy of Science. A follow-on 2019–2020 analysis by the Antimicrobial Resistance National Coordination Center also found high proportions of *Escherichia coli* and *Staphylococcus aureus* clinical isolates resistant to commonly used antibiotics [4, 5].

In sentinel surveillance at clinical sites in Uganda, the use of antimicrobials is driven by availability, access, and affordability, and not necessarily by efficacy and suitability [5]. Furthermore, a retrospective cross-sectional study from lower-level health centers in Mbarara district showed 75% unnecessary prescriptions for treating upper respiratory tract infections in children aged under 5 years in ambulatory care [6]. Both reports acknowledge the limited public and health worker awareness of excessive antimicrobial use as a major contributor to AMR and indicate that a better understanding is needed of how confounding factors, like limited diagnostics, contribute. Antimicrobial stewardship practices can reduce the development and spread of AMR [2, 7].

However, stewardship is complicated by the fact that most antibiotic use is empiric, without confirmation of whether or not a febrile illness is caused by bacterial infection, which leads to the prescription of “just-in-case” antibiotics [7]. Such scenarios are common in health facilities in low- and middle-income countries (LMICs) like Uganda, especially in outpatient settings, where diagnostics to identify common causes of febrile illnesses other than malaria are lacking. This dilemma could be improved by the use of rapid point-of-care tests (POCTs) to help distinguish bacterial infections from other causes and to guide antibiotic use [7]. In HIV/AIDS and malaria, 2 pandemics that have ravaged LMICs, the use of POCTs facilitates testing for millions of people due to low cost and universal accessibility, and allows targeted antimicrobial treatment [8, 9].

We investigated whether adopting a panel of commercially available POCTs intended to help clinicians distinguish between bacterial and other infectious causes of acute febrile illness, combined with clinical diagnostic algorithms and behavior change activities, could safely reduce antibiotic prescribing compared with current standard-of-care for febrile patients presenting to outpatient clinics in Uganda.

## METHODS

### Objectives, Endpoints, and Assessments

The study goal was to assess the impact of the intervention compared with standard-of-care practices on antibiotic prescribing and clinical outcomes for outpatients presenting with nonsevere acute febrile illness. Primary endpoints were the proportion of patients who received an antibiotic prescription and the proportion who had a favorable clinical outcome (defined at day 7 as having a normal body temperature and reporting that day 0 symptoms had improved or resolved). Prespecified subgroup analyses of the primary endpoints were conducted for age, gender, and enrollment period. Other subgroup analyses such as malaria rapid diagnostic test (mRDT) result and respiratory syndromes were exploratory.

### Study Design

This study was part of a multicountry project with harmonized research protocols. In Uganda, we conducted an open-label, 2-arm, multicenter, randomized controlled trial. Study participants were randomized to either an intervention or control group using a 1:1 scheme using varying block sizes of 64, 96, or 128 in random order. Randomization lists for each health facility, which included consecutive identification numbers with corresponding random arm assignments, were computer-generated from the FIND Geneva. Randomized codes that corresponded to the 2 (intervention and control) arms were also generated using permuted variable-sized blocks. At each study site, each consecutive identification number with its corresponding treatment code was placed in a sealed envelope and kept in that order. Each consecutive participant was assigned an ID number and study arm from the next available envelope during randomization.

### Study Sites

Three public health facilities were selected to represent different geographical and cultural regions of Uganda and different malaria transmission intensities, which were anticipated to influence prescribing practices for acute febrile illnesses: Aduku Health Centre (HC) IV in Kwania District in the north, a region of high malaria transmission; Nagongera HC IV in Tororo District in the east, where transmission historically was high but in recent years is considered low transmission due to regular indoor residual spraying; and Kihihi HC IV in Kanungu District in the southwest, with moderate transmission [10, 11]. In the Ugandan public health care system, health center IVs provide the second highest level of care, between hospitals and more basic outpatient clinics. Health center IVs typically offer outpatient, inpatient, and maternity care for catchment areas that include rural and semi-urban populations. Staff typically include clinicians who have from 1.5 to 5 years of formal training (1 or 2 medical officers or clinical officers and nursing staff), and laboratory staff who perform malaria, HIV, and syphilis rapid diagnostic tests (RDTs); malaria microscopy; hemoglobin estimation; CD4 count; urinalysis; and sputum microscopy, depending on the availability of materials [12].

### Participants

The study participants included patients aged 1 year and older who presented to the outpatient departments of the participating health centers with acute febrile illness, defined as tympanic temperature of more than 37.5°C or axillary temperature of more than 37.0°C or history of fever lasting 7 days or fewer, with no focus or with a suspected respiratory tract infection. Patients with signs or symptoms suggestive of severe disease who required hospitalization or referral and those with skin/soft tissue infection as a probable cause of fever were excluded.

### Intervention

**Figure 1.**
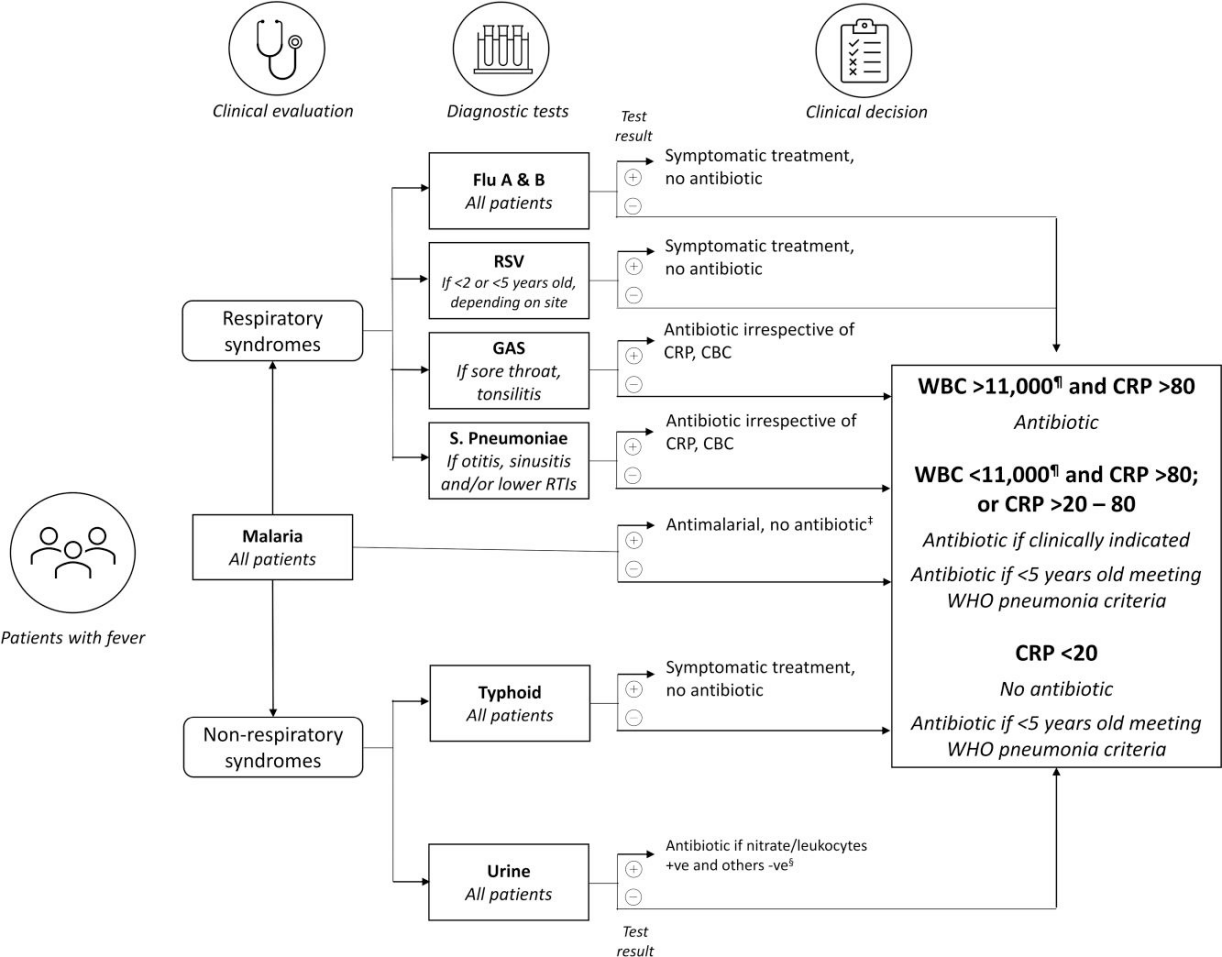
Diagnostic and treatment algorithm for the intervention arm of a randomized controlled trial that introduced point-of-care diagnostic tests to guide the management of outpatients with febrile illness in Uganda. ^‡^Unless a concomitant bacterial pathogen identified. ^§^Start treatment followed by culture if needed. ^¶^And neutrophils >75% if WBC >11 000 and/or neutrophils >75% if WBC <11 000. Abbreviations: CBC, complete blood count; CRP, C-reactive protein (mg/L); GAS, group A streptococci; RSV, respiratory syncytial virus; RTI, respiratory tract infection; WBC, white blood cell count (per μL); WHO, World Health Organization.

The intervention had 3 components: a specified panel of POCTs; a diagnostic and treatment algorithm (Figure 1) that guided clinicians on selecting tests to perform for each participant, and on deciding which patients needed an antibiotic prescription; and a training-and-communication (T&C) package of messages geared towards health workers and patients. For the health workers, the T&C package was designed to help them communicate better with patients about adherence to prescriptions; the messages for patients were meant to encourage adherence to the prescription received. The T&C package was developed from social science work conducted before the clinical trial began and is described in another article in this supplement issue (Kaawa-Mafigiri et al).

### Study Procedures

Before enrollment, each patient provided written informed consent to participate in this study. For children aged 7 years and younger, a parent/guardian provided consent on their behalf. Children aged 8–17 years provided assent and their parent/guardian gave consent as well. Patients 18 years and older provided informed consent for themselves.

For each of the 3 study sites, throughout the study all patients randomized to the intervention arm were seen by a clinical officer (prescriber) and a laboratory technologist hired by the study team. The intervention-arm prescribers and laboratory technologists were trained on aspects of the protocol—including informed-consent procedures, enrollment, randomization, how to use the diagnostic and treatment algorithm, how to use the POCTs, and delivering the T&C package—for 1 week prior to enrollment, and study co-investigators reinforced training messages and addressed questions during regular site visits and communication with intervention-arm staff during the course of the study. Patients randomized to the control arm received the standard-of-care from clinical officers (usually 1 to 3 in number depending on staffing norms at each site and on each day) and laboratory staff employed by the participating health facilities, who requested and performed laboratory testing and prescribed medicines according to their routine standard practice. The control-arm staff were aware that the study was being conducted but were not trained in intervention-arm procedures.

The day of enrollment was designated as day 0. Eligible patients who consented were enrolled into the study and randomized to either the intervention arm or the control arm. In both study arms, vital signs were measured and a clinician took a history of the patient's illness, performed a physical exam, and recorded a preliminary diagnosis. In the intervention arm, the clinician applied the diagnostic and treatment algorithm (Figure 1) to decide which POCTs to request from the laboratory, except for the mRDT, which was performed for all patients in this arm in line with national guidelines.

Point-of-care tests for the intervention arm included the following: Test-it Typhoid fever IgM rapid test kit (Life Assay Diagnostics); SD Bioline influenza Antigen A/B/A (H1N1) pandemic rapid test kit (Alere/Abbott); Alere BinaxNow respiratory syncytial virus (RSV) card; Streptococcus A test kit (Sekisui OSOM); *Streptococcus pneumoniae* urine antigen card test (Abbott/Alere); 5-part white blood cell (WBC) differential count POCT (HemoCue); 10-parameter urine test strips (Multistix 10 SG) for leucocyte esterase and nitrites (Siemens); Standard F C-reactive protein (CRP) (SD Biosensor). The POCT results in the intervention arm were typically available within 30 minutes after referral to the laboratory and were used by the clinician to make prescribing decisions.

Patients in both study arms received prescribed medicines free of charge from the dispensary at each health center, according to the government supplies and standard practice. All patients were asked to return to the health center 7 days after enrollment for assessment of the outcome of their illness and of adherence to the day 0 prescription. Adherence to prescription was assessed through pill counts conducted by study clinicians and in-depth interviews conducted by social science research assistants.

### Sample Size and Statistical Analysis

An optimal sample size of 2400 was determined based on the expected relative reduction in antibiotic prescriptions of 30%, at 80% power and a significance level of 5% [13]. Our primary interest was the overall population-level effect by the intention-to-treat principle. Therefore, primary hypothesis testing related to between-arm comparison was done at the nominal α-level (.05).

In addition, several subgroup analyses were performed. Due to the explorative character of subgroup analyses (those prespecified in statistical analysis plan, and those arising from observed data patterns), emphasis is laid on the clinical relevance of the observed effects. Statistical significance assessment for within-subgroup comparisons was done without adjustment for multiple test situations, consistently providing 95% confidence intervals (CIs) for obtained point estimates.

Descriptive statistics tables were generated to summarize the characteristics of the patients. The information was categorized by site, gender, and age group. Results were reported either in absolute numbers (eg, number of subjects in a group) or summarized by mean, median, standard deviation, minimum, maximum, and quartiles, as appropriate. Data analysis was performed using R version 4.2.1 (R Foundation for Statistical Computing).

### Ethics Statement

The study protocol was approved by the Makerere University School of Biomedical Sciences Higher Degrees Research and Ethics Committee (SBS-REC reference SBS715), the Uganda National Council for Science and Technology (reference HS 2727), Oxford Tropical Research Ethics Committee (OxTREC reference 52-19) and the London School of Hygiene and Tropical Medicine Research Ethics Committee (reference 26684).

## RESULTS

**Figure 2.**
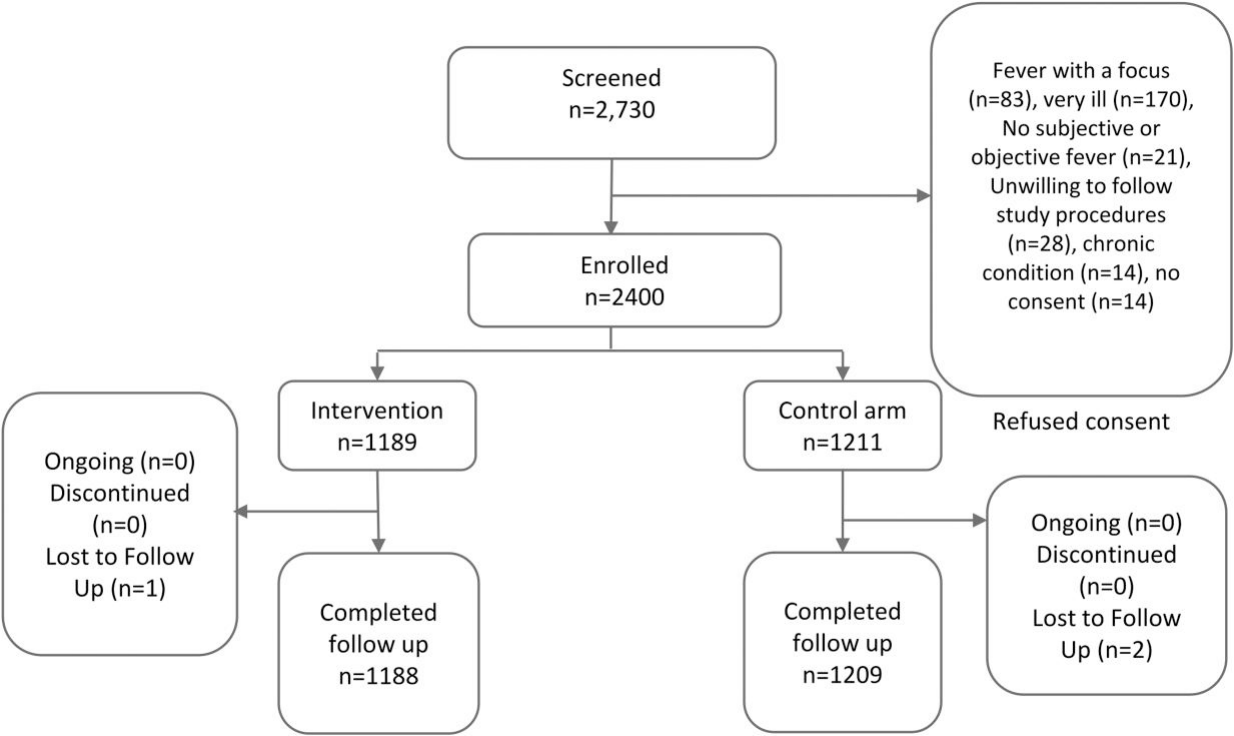
Participant disposition.

A total of 2400 patients were enrolled across the 3 sites, 800 at each site. Of the 2400, 49.5% were randomized to the intervention arm. Figure 2 shows patient recruitment and disposition.

### Baseline Demographics and Characteristics

Across all sites, over half of patients were female and approximately one-third (30.9%) were children aged 1 to 5 years. Nearly half (46.5%) of the patients presented with respiratory symptoms (Table 1).

**Table 1.** Baseline Characteristics of Participants in a Randomized Controlled Trial That Introduced Point-of-Care Diagnostic Tests to Guide Management of Outpatients With Febrile Illness, Conducted at 3 Clinical Sites in Uganda

Characteristics	All Sites		Aduku (Kwania District)		Nagongera (Tororo District)		Kihihi (Kanungu District)	
	Intervention	Control	Intervention	Control	Intervention	Control	Intervention	Control
Total enrolled, n (%)	1189 (49.5%)	1211 (50.5%)	401 (50.1%)	399 (49.9%)	398 (49.8%)	402 (50.2%)	390 (48.8%)	410 (51.2%)
Patient age, n (%)								
** **<5 years	364 (30.6%)	377 (31.1%)	116 (28.9%)	67 (16.8%)	158 (39.7%)	198 (49.3%)	90 (23.1%)	112 (27.3%)
** **5 to <10 years	196 (16.5%)	236 (19.5%)	60 (15.0%)	83 (20.8%)	69 (17.3%)	66 (16.4%)	67 (17.2%)	87 (21.2%)
** **≥10 to <15 years	102 (8.6%)	111 (9.2%)	33 (8.2%)	43 (10.8%)	23 (5.8%)	21 (5.2%)	46 (11.8%)	47 (11.5%)
** **≥15 years	527 (44.3%)	487 (40.2%)	192 (47.9%)	206 (51.6%)	148 (37.2%)	117 (29.1%)	187 (48.0%)	164 (40.0%)
Patient age, median (Q1, Q3), y	11 (4, 23)	9 (3, 20)	13 (4, 23.5)	15 (6, 21)	6 (3, 22)	5 (2, 18)	13.5 (5, 23)	10.5 (4, 20)
Patient gender, n (%)								
** **Female	717 (60.3%)	742 (61.3%)	269 (67.1%)	252 (63.2%)	226 (56.8%)	243 (60.5%)	222 (56.9%)	247 (60.2%)
** **Male	472 (39.7%)	469 (38.7%)	132 (32.9%)	147 (36.8%)	172 (43.2%)	159 (39.6%)	168 (43.1%)	163 (39.8%)
Date of enrollment, n (%)								
** **July–September 2020	19 (1.6%)	21 (1.7%)	7 (1.8%)	7 (1.8%)	12 (3.0%)	14 (3.5%)	0 (0.0%)	0 (0.0%)
** **October–December 2020	258 (21.7%)	256 (21.1%)	88 (22.0%)	84 (21.1%)	88 (22.1%)	85 (21.1%)	82 (21.0%)	87 (21.2%)
** **January–March 2021	326 (27.4%)	340 (28.1%)	114 (28.4%)	120 (30.1%)	101 (25.4%)	100 (24.9%)	111 (28.5%)	120 (29.3%)
** **April–June 2021	389 (32.7%)	375 (31.0%)	136 (33.9%)	124 (31.1%)	131 (32.9%)	132 (32.8%)	122 (31.3%)	119 (29.0%)
** **July–September 2021	197 (16.6%)	219 (18.1%)	56 (14.0%)	64 (16.0%)	66 (16.6%)	71 (17.7%)	75 (19.2%)	84 (20.5%)
Respiratory vs nonrespiratory syndrome,^a^ n (%)								
** **Respiratory	648 (54.5%)	421 (34.8%)	206 (51.4%)	179 (44.9%)	255 (64.1%)	183 (50.1%)	187 (48.0%)	59 (14.4%)
** **Nonrespiratory	541 (45.5%)	753 (62.2%)	195 (48.6%)	220 (55.1%)	143 (35.9%)	182 (49.9%)	203 (52.1%)	351 (85.6%)

Abbreviation: Q, quarter.

As indicated by the managing clinician based on symptoms and signs, before performance of diagnostic tests. Note this information was not recorded on 37 patient forms in the Nagongera control arm.

### Point-of-Care Diagnostic Tests

**Figure 3.**
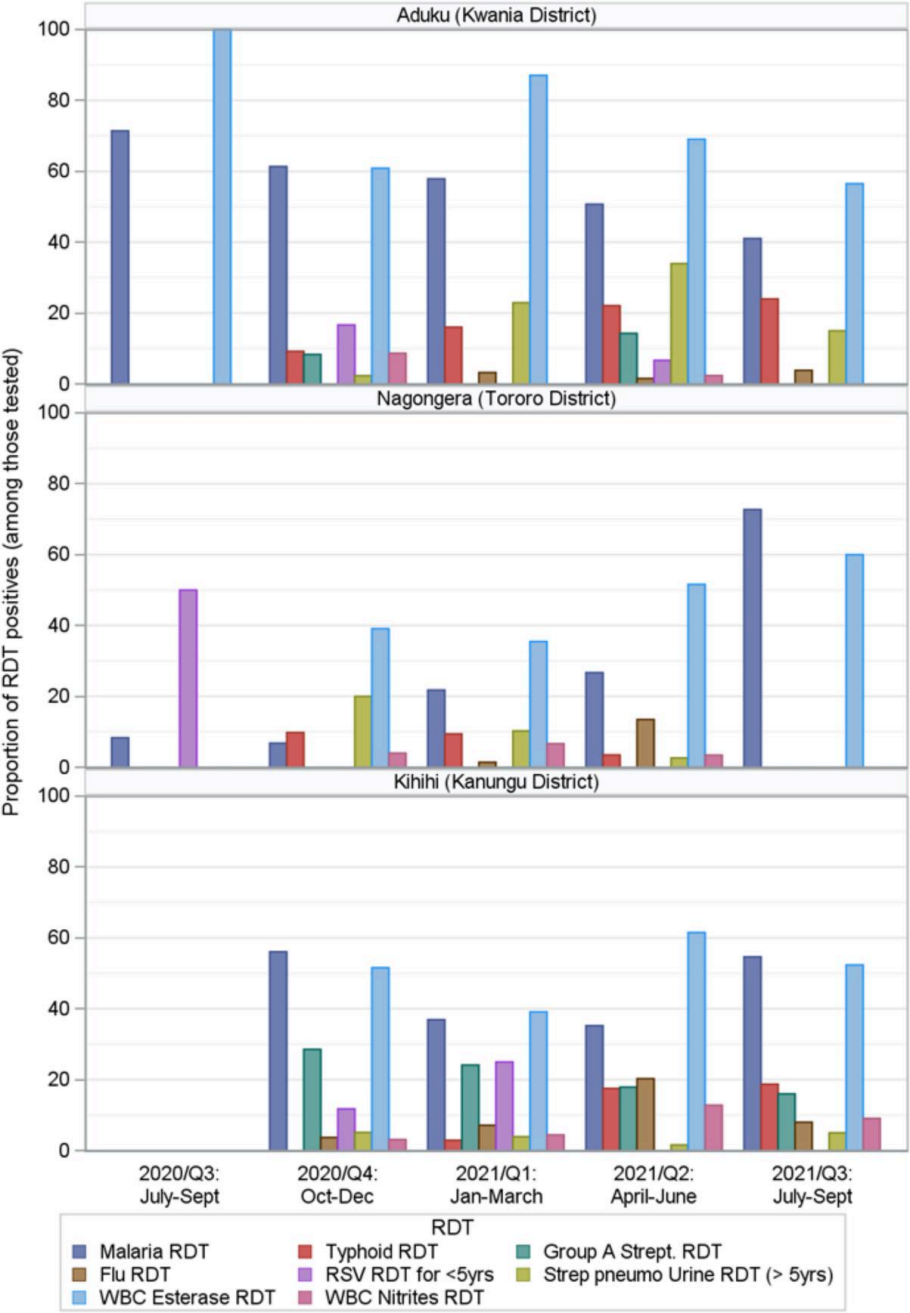
Bar chart showing the proportion positive of POCTs performed in the intervention arm, over the full study period, in a randomized controlled trial that introduced POCTs to guide the management of outpatients with febrile illness at 3 clinical sites in Uganda. The proportion of each POCT with positive results is shown on the *y*-axis and months of the year on the *x*-axis. Abbreviations: POCT, point-of-care test; RDT, rapid diagnostic test.

Table 2 and Figure 3 show the POCTs performed and their results. In the control arms at all 3 sites, few diagnostic tests were done except for malaria testing. In the intervention arms, approximately 10% of patients tested had an elevated WBC count and/or differential in Aduku and Kihihi (11.3% and 10.5%, respectively), while this proportion was 18.3% in Nagongera. At all 3 sites, CRP levels were elevated (20–80 mg/L or >80 mg/L) in approximately one-third of intervention-arm patients tested. All intervention-arm patients were tested for malaria with mRDT, with positivity ranging from 28.1% in Nagongera to 43.9% in Kihihi to 54.1% in Aduku. For other pathogen-specific tests, the numbers tested and proportions positive varied across the sites (Table 2).

**Table 2.** Point-of-Care Tests Performed for Participants in the Intervention Arm of a Randomized Controlled Trial That Introduced Point-of-Care Diagnostic Tests to Guide Management of Outpatients With Febrile Illness at 3 Clinical Sites in Uganda

	All 3 Ugandan Sites (N = 1189)		Aduku (n = 401)		Nagongera (n = 398)		Kihihi (n = 390)	
Test	Performed	Positive	Performed	Positive	Performed	Positive	Performed	Positive
Pathogen-specific POCTs								
** **Malaria	1189 (100%)	500 (42.1%)	401 (100%)	217 (54.1%)	398 (100%)	112 (28.1%)	390 (100%)	171 (43.8%)
** **Typhoid	781 (65.7%)	92 (11.8%)	327 (81.5%)	58 (17.7%)	239 (60.0%)	13 (5.4%)	215 (55.1%)	21 (9.8%)
** **Group A *Streptococcus*	285 (24.0%)	48 (16.8%)	29 (7.2%)	2 (6.9%)	50 (12.6%)	0 (0.0%)	206 (52.8%)	46 (22.3%)
** **Influenza A/B/A	681 (57.3%)	39 (5.7%)	211 (52.6%)	4 (1.9%)	266 (66.8%)	13 (4.9%)	204 (52.3%)	22 (10.8%)
** **RSV (<5 y)	141 (11.9%)	8 (5.7%)	34 (8.5%)	2 (5.9%)	65 (16.3%)	1 (1.5%)	42 (10.7%)	5 (11.9%)
** ** *Streptococcus pneumoniae* (>5 y)	470 (39.5%)	48 (10.2%)	166 (41.4%)	33 (19.9%)	131 (32.9%)	9 (6.9%)	173 (44.3%)	6 (3.5%)
PoC biomarker tests								
** **Urine leukocyte esterase	354 (29.8%)	195 (55.1%)	120 (29.9%)	84 (70.0%)	97 (24.4%)	42 (43.3%)	137 (35.1%)	69 (50.4%)
** **Urine nitrites	352 (29.6%)	17 (4.8%)	120 (29.9%)	3 (2.5%)	94 (23.6%)	4 (4.3%)	138 (35.4%)	10 (7.3%)
								
Test	Done n(%)	Median (Q1, Q3)	Done n(%)	Median (Q1, Q3)	Done n(%)	Median (Q1, Q3)	Done n(%)	Median (Q1, Q3)
CRP, mg/L	1188 (99.9%)	6.0 (1.0, 35.3)	400 (99.8%)	2.0 (1.0, 46.7)	398 (100%)	7.1 (1.0, 71.9)	390 (100%)	8.4 (1.0, 27.7)
WBC counts (×1000)	1187 (99.8%)	6.8 (5.0, 9.2)	400 (99.8%)	6.9 (5.1, 9.1)	398 (100%)	7.4 (5.4, 9.9)	389 (99.7%)	6.1 (4.6, 8.6)
Neutrophil counts, %	1186 (99.7%)	40 (32, 50)	399 (99.5%)	38 (29, 47)	398 (100%)	40 (31, 51)	389 (99.7%)	44 (35, 54)
	No.	Proportion, %	No.	Proportion, %	No.	Proportion, %	No.	Proportion, %
CRP <20 mg/L	770	64.8%	264	66.0%	253	63.6%	253	64.9%
CRP 20–80 mg/L	197	16.6%	50	12.5%	47	11.8%	100	25.6%
CRP >80 mg/L	221	18.6%	86	21.5%	98	24.6%	37	9.5%
WBC count <11 000	1030	86.8%	354	88.5%	325	81.7%	351	90.2%
WBC count ≥11 000	157	13.2%	46	11.5%	73	18.3%	38	9.8%

Data are presented as n (%) unless otherwise indicated.

Abbreviations: CRP, C-reactive protein; PoC, point-of-care; POCT, point-of-care test; Q, quartile; RSV, respiratory syncytial virus; WBC, white blood cell.

### Antibiotic Prescribing

Overall, there was no difference in antibiotic prescriptions between arms, with a relative risk (RR) of 1.03 (95% CI: .96–1.11); however, the size and direction of effects varied across sites and in different subpopulations (Table 3). Across the 3 sites, only Nagongera showed a marginally statistically significant, but small difference between study arms, with an RR of 1.19 (1.00–1.41) greater in the intervention arm, whereas no difference was seen for Aduku (1.04; 95% CI: .93–1.16) and Kihihi (.94; 95% CI: .85–1.05). In age-stratified analysis, for children (<5, 5–10, and 10–15 years) in Aduku and Nagongera, larger differences in favor of more antibiotic prescription were seen, although this was not significant in all subgroups (1.43 [1.01–1.85], 1.81 [1.28–2.58], 1.38 [0.83–2.31]; and 1.37 [1.05–1.77], 1.58 [.96–2.60], 2.51 [.94, 6.69], respectively). For older patients in these sites, inverse results were observed (RR: .74 [.64–0.85] and .82 [.63–1.05], respectively). In Kihihi, the situation was different: antibiotic prescribing was lower in children aged younger than 5 years (RR: .76; 95% CI: .62–.94) but was not significantly different in older age groups.

**Table 3.** Proportion of Participants Who Were Prescribed Any Antibiotic in a Randomized Controlled Trial That Introduced Point-of-Care Tests to Guide Management of Outpatients With Febrile Illness at 3 Clinical Sites in Uganda

	All Sites			Aduku (Kwania District)			Nagongera (Tororo District)			Kihihi (Kanungu District)		
Antibiotic Prescription	Intervention, n/N (%)	Control, n/N (%)	RR [95% CI]	Intervention, n/N (%)	Control, n/N (%)	RR [95% CI]	Intervention, n/N (%)	Control, n/N (%)	RR [95% CI]	Intervention, n/N (%)	Control, n/N (%)	RR [95% CI]
Total (all ages)	661/1188 (55.6%)	654/1211 (54.0%)	1.03 [.96, 1.11]	248/401 (61.8%)	238/399 (59.6%)	1.04 [.93, 1.16]	174/398 (43.7%)	148/402 (36.8%)	1.19 [1.00, 1.41]	239/389 (61.4%)	268/410 (65.4%)	.94 [.85, 1.05]
Patient age categories												
** **<5 years	208/364 (57.1%)	185/377 (49.1%)	1.16 [1.02, 1.33]	84/116 (72.4%)	34/67 (50.7%)	1.43 [1.10, 1.85]	72/158 (45.6%)	66/198 (33.3%)	1.37 [1.05, 1.77]	52/90 (57.8%)	85/112 (75.9%)	.76 [.62, .94]
** **5 to <10 years	109/196 (55.6%)	94/236 (39.8%)	1.40 [1.14, 1.71]	38/60 (63.3%)	29/83 (34.9%)	1.81 [1.28, 2.58]	28/69 (40.6%)	17/66 (25.8%)	1.58 [.96, 2.60]	43/67 (64.2%)	48/87 (55.2%)	1.16 [.9, 1.51]
** **10 to <15 years	56/102 (54.9%)	46/111 (41.4%)	1.33 [1.00, 1.76]	17/33 (51.5%)	16/43 (37.2%)	1.38 [.83, 2.31]	11/23 (47.8%)	4/21 (19.0%)	2.51 [.94, 6.69]	28/46 (60.9%)	26/47 (55.3%)	1.10 [.78, 1.56]
** **≥15 years	288/526 (54.8%)	329/487 (67.6%)	.81 [.73, .90]	109/192 (56.8%)	159/206 (77.2%)	.74 [.64, .85]	63/148 (42.6%)	61/117 (52.1%)	.82 [.63, 1.05]	116/186 (62.4%)	109/164 (66.5%)	.94 [.80, 1.10]
Gender												
** **Female	396/716 (55.3%)	411/742 (55.4%)	1.00 [.91, 1.10]	171/269 (63.6%)	159/252 (63.1%)	1.01 [.88, 1.15]	96/226 (42.5%)	97/243 (39.9%)	1.06 [.86, 1.32]	129/221 (58.4%)	155/247 (62.8%)	.93 [.80, 1.08]
** **Male	265/472 (56.1%)	243/469 (51.8%)	1.08 [.96, 1.22]	77/132 (58.3%)	79/147 (53.7%)	1.09 [.88, 1.34]	78/172 (45.3%)	51/159 (32.1%)	1.41 [1.07, 1.87]	110/168 (65.5%)	113/163 (69.3%)	.94 [.81, 1.10]
Enrollment period												
** **July–September 2020	5/19 (26.3%)	14/21 (66.7%)	.40 [.18, .89]	2/7 (28.6%)	4/7 (57.1%)	.50 [.13, 1.90]	3/12 (25.0%)	10/14 (71.4%)	.35 [.12, .99]	0/0	0/0	…
** **October–December 2020	129/58 (50.0%)	146/256 (57.0%)	.88 [.75, 1.03]	42/88 (47.7%)	48/84 (57.1%)	.84 [.63, 1.11]	32/88 (36.4%)	47/85 (55.3%)	.66 [.47, 0.92]	55/82 (67.1%)	51/87 (58.6%)	1.14 [.91, 1.44]
** **January–March 2021	195/325 (60.0%)	188/340 (55.3%)	1.09 [.95, 1.24]	77/114 (67.5%)	69/120 (57.5%)	1.18 [.96, 1.43]	41/101 (40.6%)	36/100 (36.0%)	1.13 [.79, 1.60]	77/110 (70.0%)	83/120 (69.2%)	1.01 [.85, 1.20]
** **April–June 2021	227/389 (58.%)	207/375 (55.2%)	1.06 [.93, 1.20]	102/136 (75.0%)	76/124 (61.3%)	1.22 [1.03, 1.45]	59/131 (45.0%)	39/132 (29.5%)	1.52 [1.10, 2.11]	66/122 (54.1%)	92/119 (77.3%)	0.70 [.58, .85]
** **July–September 2021	105/197 (53.3%)	99/219 (45.2%)	1.18 [.97, 1.43]	25/56 (44.6%)	41/64 (64.1%)	.70 [.49, .98]	39/66 (59.1%)	16/71 (22.5%)	2.62 [1.63, 4.22]	41/75 (54.7%)	42/84 (50.0%)	1.09 [.81, 1.47]
Respiratory vs nonrespiratory syndrome^a^												
** **Respiratory	349/648 (53.9%)	223/421 (53.0%)	1.02 [.91, 1.14]	132/206 (64.1%)	116/179 (64.8%)	1.00 [.85, 1.15]	103/255 (40.4%)	58/183 (31.7%)	1.27 [.98, 1.65]	114/187 (61.0%)	49/59 (83.1%)	.73 [.62, .86]
** **Nonrespiratory	312/540 (57.8%)	412/753 (54.7%)	1.06 [.96, 1.16]	116/195 (59.5%)	122/220 (55.5%)	1.07 [.91, 1.27]	71/143 (49.7%)	71/182 (39.0%)	1.27 [1.00, 1.63]	125/202 (61.9%)	219/351 (62.4%)	.99 [.87, 1.14]
Malaria RDT result												
** **Positive	313/500 (62.6%)	170/473 (35.9%)	1.74 [1.52, 2.00]	138/217 (63.6%)	80/189 (42.3%)	1.50 [1.24, 1.83]	72/112 (64.3%)	11/96 (11.5%)	5.61 [3.16, 9.95]	103/171 (60.2%)	79/188 (42.0%)	1.43 [1.17, 1.76]
** **Negative	348/688 (50.6%)	376/508 (74.0%)	.68 [.63, .75]	110/184 (59.8%)	121/144 (84.0%)	.71 [.62, .82]	102/286 (35.7%)	75/163 (46.0%)	.78 [.62, .97]	136/218 (62.4%)	180/201 (89.6%)	.70 [.62, .78]

Abbreviations: CI, confidence interval; RDT, rapid diagnostic test; RR, relative risk.

As indicated by the managing clinician based on symptoms and signs, before performance of diagnostic tests.

No significant differences in antibiotic prescribing were observed between study arms for patients who presented with respiratory symptoms, or for those who presented without respiratory symptoms and signs (Table 3).

In contrast, malaria status was significantly associated with prescribing at all 3 sites. In the intervention arm, patients with positive mRDTs had a higher RR of being prescribed antibiotics than those with negative mRDT results (Table 3). A possible explanation for this observation is shown in Tables 4, 5 and 6: Compared with prescribing that would have resulted from strict adherence to the algorithm, in the intervention arm, 60.1% (188/313) of malaria-positive patients and 13.5% (47/348) of malaria-negative patients were incorrectly prescribed an antibiotic (Table 5). However, if elevated CRP and/or complete blood count (CBC) and differential results had been prioritized (contrary to a strict interpretation of the algorithm that disregarded CRP and CBC results in cases where the mRDT was positive), just 2.9% (9/313) of malaria-positive patients and 11.5% (40/348) of malaria-negative patients incorrectly received an antibiotic prescription (Table 6). Among patients who tested negative by mRDT, there was a significant reduction in antibiotic prescriptions (RR: .68; 95% CI: .63–.75). These patterns were consistent across all 3 sites with varied malaria endemicity (Table 3).

**Table 4.** Prescribing That Should Have Resulted if the Diagnostic and Treatment Algorithm Were Strictly Followed, for Intervention-Arm Patients by Malaria Rapid Diagnostic Test Result

Prescribing Guidance if Algorithm Were Strictly Followed	All Intervention-Arm Patients With mRDT Results		All Sites Combined mRDT Results				Aduku (Kwania District) mRDT Results				Nagongera (Tororo District) mRDT Results				Kihihi (Kanungu District) mRDT Results			
			Neg		Pos		Neg		Pos		Neg		Pos		Neg		Pos	
	n	%	n	%	n	%	n	%	n	%	n	%	n	%	n	%	n	%
Don’t prescribe antibiotic	638	53.7	269	39.0	369	73.8	63	34.2	140	64.5	121	42.3	104	92.9	85	38.8	125	73.1
Prescribe antibiotic if clinically indicated^a^	175	14.7	175	25.4	0	0.0	34	18.5	0	0.0	94	32.9	0	0.0	47	21.5	0	0.0
Prescribe antibiotic	376	31.6	245	35.6	131	26.2	87	47.3	77	35.5	71	24.8	8	7.1	87	39.7	46	26.9
Total	1189	100	689	100	500	100	184	100	217	100	286	100	112	100	219	100	171	100

Abbreviations: mRDT, malaria rapid diagnostic test; Neg, negative; Pos, positive.

If clinically indicated in the clinician's judgment and/or if the patient was aged <5 years and met World Health Organization (WHO) pneumonia criteria.

**Table 5.** Prescribing That Should Have Resulted if the Diagnostic and Treatment Algorithm Were Strictly Followed, for Intervention-Arm Patients by Malaria Rapid Diagnostic Test Result, Among Patients Who Actually Received an Antibiotic Prescription and Those Who Did Not

Prescribing Guidance if Algorithm Were Strictly Followed	All mRDTs		All Sites				Aduku (Kwania District)				Nagongera (Tororo District)				Kihihi (Kanungu District)			
			Neg		Pos		Neg		Pos		Neg		Pos		Neg		Pos	
	n	%	n	%	n	%	n	%	n	%	n	%	n	%	n	%	n	%
Received antibiotic prescription																		
Don’t prescribe antibiotic	235	35.6	47	13.5	188	60.1	2	1.8	61	44.2	9	8.8	64	88.9	36	26.5	63	61.2
Prescribe antibiotic if clinically indicated^a^ or if CRP and/or CBC elevated	68	10.3	68	19.5	0	0.0	21	19.1	0	0.0	23	22.5	0	0.0	24	17.6	0	0.0
Prescribe antibiotic	358	54.2	233	67.0	125	39.9	87	79.1	77	55.8	70	68.6	8	11.1	76	55.9	40	38.8
Total	661	100	348	100	313	100	110	100	138	100	102	100	72	100	136	100	103	100
Did not receive antibiotic prescription																		
Don’t prescribe antibiotic	403	76.5	222	65.3	181	96.8	61	82.4	79	100	112	60.9	40	100	49	59.8	62	91.2
Prescribe antibiotic if clinically indicated^a^ or if CRP and/or CBC elevated	107	20.3	107	31.5	0	0.0	13	17.6	0	0.0	71	38.6	0	0.0	23	28.0	0	0.0
Prescribe antibiotic	17	3.2	11	3.2	6	3.2	0	0.0	0	0.0	1	0.5	0	0.0	10	12.2	6	8.8
Total	527	100	340	100	187	100	74	100	79	100	184	100	40	100	82	100	68	100

Among those who actually received an antibiotic prescription, the top row (“Don’t prescribe antibiotic”; bold values), shows over-prescription (ie, patients for whom strict adherence to the algorithm would have resulted in no antibiotic, but who in fact were prescribed an antibiotic). Among those who did not receive an antibiotic prescription, the third row (“Prescribe antibiotic”, bold values) shows under-prescription (ie, patients for whom strict adherence to the algorithm would have resulted in an antibiotic, but who in fact were not prescribed one).

Abbreviations: CBC, complete blood count; CRP, C-reactive protein; mRDT, malaria rapid diagnostic test; Neg, negative; Pos, positive.

If clinically indicated in the clinician's judgment and/or if the patient was aged <5 years and met World Health Organization (WHO) pneumonia criteria.

**Table 6.** Prescribing That Would Have Resulted if the Diagnostic and Treatment Algorithm Were Modified So That CRP and/or CBC and Differential Were Prioritized for All Patients Regardless of Malaria Status, for Intervention-Arm Patients by Malaria Rapid Diagnostic Test Result, Among Patients Who Actually Received an Antibiotic Prescription and Those Who Did Not

Prescribing Guidance if CRP and/or CBC Results Were Prioritized for All Patients	All mRDTs		All Sites				Aduku (Kwania District)				Nagongera (Tororo District)				Kihihi (Kanungu District)			
			Neg		Pos		Neg		Pos		Neg		Pos		Neg		Pos	
	n	%	n	%	n	%	n	%	n	%	n	%	n	%	n	%	n	%
Received antibiotic prescription																		
Don’t prescribe antibiotic	49	7.4	40	11.5	9	2.9	2	1.8	0	0.0	7	6.9	0	0.0	31	22.8	9	8.7
Prescribe antibiotic if clinically indicated^a^ or if CRP and/or CBC elevated	226	34.2	74	21.3	152	48.6	21	19.1	53	38.4	24	23.5	48	66.7	29	21.3	51	49.5
Prescribe antibiotic	386	58.4	234	67.2	152	48.6	87	79.1	85	61.6	71	69.6	24	33.3	76	55.9	43	41.7
Total	661	100	348	100	313	100	110	100	138	100	102	100	72	100	136	100	103	100
Did not receive antibiotic prescription																		
Don’t prescribe antibiotic	303	57.5	215	63.2	88	47.1	60	81.1	45	57.0	108	58.7	13	32.5	47	57.3	30	44.1
Prescribe antibiotic if clinically indicated^a^ or if CRP and/or CBC elevated	207	39.3	114	33.5	93	49.7	14	18.9	34	43.0	75	40.8	27	67.5	25	30.5	32	47.1
Prescribe antibiotic	17	3.2	11	3.2	6	3.2	0	0.0	0	0.0	1	0.5	0	0.0	10	12.2	6	8.8
Total	527	100	340	100	187	100	74	100	79	100	184	100	40	100	82	100	68	100

Among those who actually received an antibiotic prescription, the top row (“Don’t prescribe antibiotic”; bold values), shows over-prescription (ie, patients for whom strict adherence to the algorithm would have resulted in no antibiotic, but who in fact were prescribed an antibiotic). Among those who did not receive an antibiotic prescription, the third row (“Prescribe antibiotic”, bold values) shows under-prescription (ie, patients for whom strict adherence to the algorithm would have resulted in an antibiotic, but who in fact were not prescribed one).

Abbreviations: CBC, complete blood count; CRP, C-reactive protein; mRDT, malaria rapid diagnostic test; Neg, negative; Pos, positive.

If clinically indicated in the clinician's judgment and/or if the patient was aged <5 years and met World Health Organization (WHO) pneumonia criteria.

**Figure 4.**
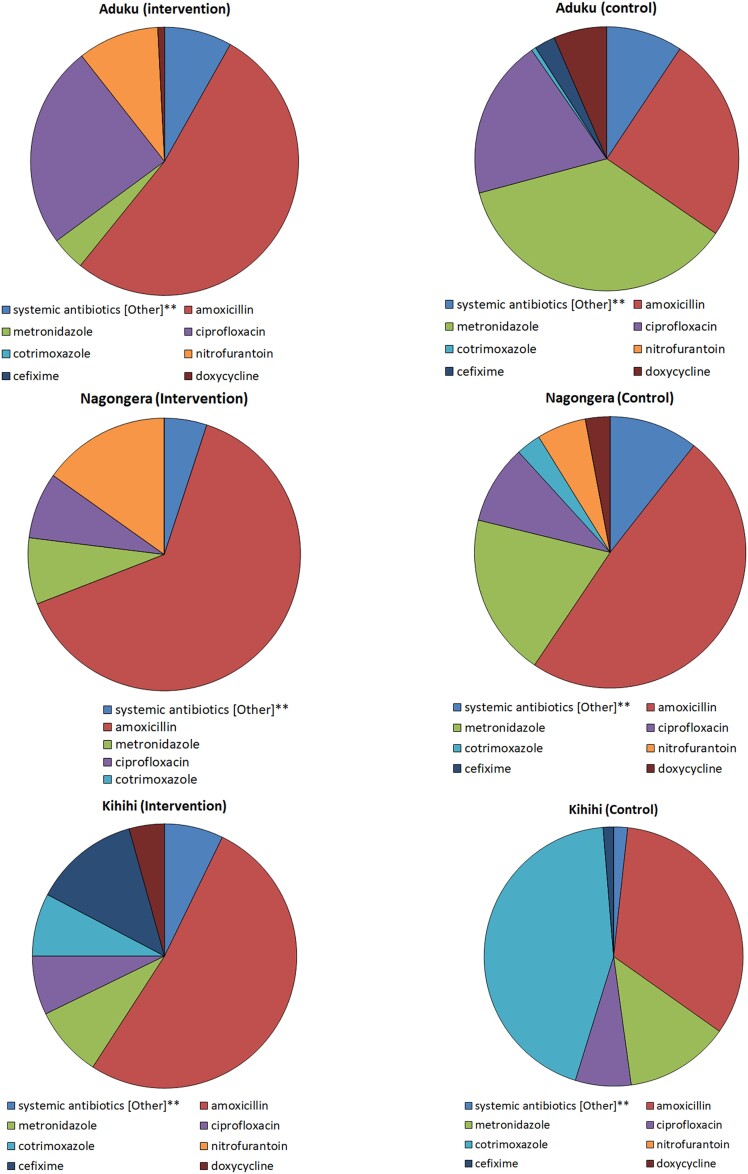
Pie charts showing the most commonly prescribed antibiotics in a randomized controlled trial that introduced point-of-care tests to guide the management of outpatients with febrile illness at 3 clinical sites in Uganda.

The most prescribed antibiotics at each site are shown in Figure 4. All sites reported prescribing systemic oral antibiotics, including amoxicillin, cefixime, doxycycline, ciprofloxacin, cotrimoxazole, metronidazole, and nitrofurantoin, among others. In the intervention arm, amoxicillin was the most-prescribed antibiotic at all 3 sites and nitrofurantoin among the top 3 prescribed antibiotics at Aduku and Nagongera, whereas in the control arm, metronidazole, amoxicillin, and cotrimoxazole accounted for the largest proportion of antibiotic prescriptions at 1 site each. Cotrimoxazole, an antibiotic not currently indicated for any of the common bacterial infections, accounted for nearly half of antibiotic prescriptions in the control arm at Kihihi, and a smaller proportion in the intervention arm.

### Clinical Outcomes

Table 7 shows the outcomes of participating patients' illnesses, stratified by selected characteristics. There was no significant difference between the intervention and control arms in clinical outcomes. There were 2 serious adverse events in the intervention arm and 1 serious adverse event in the control arm (data not shown): all 3 were children who required hospitalization for severe malaria and/or presumed septicemia, and all returned to good health by day 7.

**Table 7.** Clinical Outcome on Day 7 After Enrollment of Participants in a Randomized Controlled Trial That Introduced Point-of-Care Tests to Guide Management of Outpatients With Febrile Illness at 3 Clinical Sites in Uganda

	Aduku (Kwania District)			Nagongera (Tororo District)			Kihihi (Kanungu District)		
Clinical Outcome^a^	Intervention, n/N (%)	Control, n/N (%)	RR [95% CI]	Intervention, n/N (%)	Control, n/N (%)	RR [95% CI]	Intervention, n/N (%)	Control, n/N (%)	RR [95% CI]
Total (all ages)	30/401 (7.5%)	42/399 (10.5%)	.71 [.45, 1.11]	33/398 (8.3%)	43/402 (10.7%)	.78 [.50, 1.19]	8/390 (2.1%)	13/410 (3.2%)	.65 [.27, 1.54]
Patient age categories									
** **<5 years	10/116 (8.6%)	4/67 (6.0%)	1.44 [.47, 4.43]	14/158 (8.9%)	21/198 (10.6%)	.84 [.44, 1.59]	0/90 (0.0%)	5/112 (4.5%)	…
** **5 to <10 years	5/60 (8.3%)	7/83 (8.4%)	.99 [.33, 2.96]	7/69 (10.1%)	7/66 (10.6%)	.96 [.36, 2.58]	1/67 (1.5%)	2/87 (2.3%)	.65 [.06, 7.01]
** **10 to <15 years	1/33 (3.0%)	3/43 (7.0%)	.43 [.05, 3.99]	2/23 (8.7%)	1/21 (4.8%)	1.83 [.18, 18.70]	0/46 (0.0%)	1/47 (2.1%)	…
** **≥15 years	14/192 (7.3%)	28/206 (13.6%)	.54 [.29, .99]	10/148 (6.8%)	14/117 (12.0%)	.57 [.26, 1.23]	7/187 (3.7%)	5/164 (3.0%)	1.23 [.40, 3.79]
Gender									
** **Female	21/269 (7.8%)	32/252 (12.7%)	.62 [.36, 1.04]	14/226 (6.2%)	23/243 (9.5%)	.65 [.35, 1.24]	5/222 (2.3%)	8/247 (3.2%)	.70 [.23, 2.09]
** **Male	9/132 (6.8%)	10/147 (6.8%)	1.00 [.42, 2.39]	19/172 (11.0%)	20/159 (12.6%)	.88 [.49, 1.58]	3/168 (1.8%)	5/163 (3.1%)	.58 [.14, 2.40]
Enrollment period									
** **July–September 2020	0/7 (0.0%)	1/7 (14.3%)	…	0/12 (0.0%)	2/14 (14.3%)	…	0/0	0/0	…
** **October–December 2020	3/88 (3.4%)	8/84 (9.5%)	.36 [.10, 1.30]	7/88 (8.0%)	13/85 (15.3%)	.52 [.22, 1.24]	0/82 (0.0%)	2/87 (2.3%)	…
** **January–March 2021	8/114 (7.0%)	11/120 (9.2%)	.77 [.32, 1.83]	11/101 (10.9%)	12/100 (12.0%)	.91 [.42, 1.96]	4/111 (3.6%)	4/120 (3.3%)	1.08 [.28, 4.22]
** **April–June 2021	12/136 (8.8%)	14/124 (11.3%)	.78 [.38, 1.62]	8/131 (6.1%)	13/132 (9.8%)	.62 [.27, 1.45]	1/122 (0.8%)	6/119 (5.0%)	.16 [.02, 1.33]
** **July–September 2021	7/56 (12.5%)	8/64 (12.5%)	1.00 [.39, 2.58]	7/66 (10.6%)	3/71 (4.2%)	2.51 [.68, 9.31]	3/75 (4.0%)	1/84 (1.2%)	3.36 [.36, 31.61]
Respiratory vs nonrespiratory syndrome^b^									
** **Respiratory	16/206 (7.8%)	19/179 (10.6%)	.73 [.39, 1.38]	19/255 (7.5%)	24/183 (13.1%)	.57 [.32, 1.00]	3/187 (1.6%)	2/59 (3.4%)	.47 [.01, 2.77]
** **Nonrespiratory	14/195 (7.2%)	23/220 (10.5%)	.69 [.36, 1.30]	14/143 (9.8%)	14/182 (7.7%)	1.27 [.63, 2.58]	5/203 (2.5%)	11/351 (3.1%)	.79 [.28, 2.23]
mRDT result									
** **Positive	11/217 (5.1%)	16/189 (8.5%)	.60 [.29, 1.26]	5/112 (4.5%)	5/96 (5.2%)	.86 [.26, 2.87]	3/171 (1.8%)	5/188 (2.7%)	.66 [.16, 2.72]
** **Negative	19/184 (10.3%)	19/144 (13.2%)	.78 [.43, 1.42]	28/286 (9.8%)	19/163 (11.7%)	.84 [.49, 1.46]	5/219 (2.3%)	8/201 (4.0%)	.57 [.19, 1.73]

Numerators (n) denote unfavorable outcomes.

Abbreviations: CI, confidence interval; mRDT, malaria rapid diagnostic test; RR, relative risk.

Unfavorable outcome was defined at day 7 as an elevated body temperature and/or reporting that day 0 symptoms had not improved.

As indicated by the managing clinician based on symptoms and signs, before performance of diagnostic tests.

## DISCUSSION

This trial evaluated the impact on antibiotic prescribing and clinical outcomes, for child and adult outpatients with acute febrile illness who presented to public health centers in Uganda, of an intervention that provided diagnostic POCTs along with a case-management algorithm and behavior change materials.

A primary goal of diagnostic interventions like the one in this study is to improve antibiotic stewardship—that is, to improve targeting of antibiotics by providing them to patients with bacterial infections while reducing unnecessary use in those with nonbacterial illnesses. In this study, the overall impact on antibiotic prescribing was not consistent across sites and patient subgroups. The use of a clinical algorithm and diagnostic POCTs did not demonstrate a substantial reduction in antibiotic prescribing in the intervention arm, except in patients with a negative malaria test who were consistently prescribed fewer antibiotics. There are a few potential reasons for this.

First, our intervention introduced commercially available POCTs for specific bacterial infections (typhoid, group A *Streptococcus* and *S. pneumoniae*), specific viral infections (influenza and RSV), and a parasitic infection (malaria) as well as biomarkers associated with bacterial versus other infections (WBC and differential and CRP in blood and leukoesterase and nitrites in urine). Although these testing options represent more diagnostic capacity than is currently available in routine care, not surprisingly, the majority of results for any given test (besides malaria RDT) were negative, leaving a large proportion of fevers without a specific pathogen–based diagnosis. Experience implementing malaria RDTs in LMICs has shown that, in the absence of an alternative specific diagnosis, many clinicians continue to prescribe antibiotics “just in case” [14–16]. Meaningful improvements in antibiotic stewardship may require more time for clinicians and patients to adapt and develop confidence in new tools, with sufficient training and knowledge around the tools and their utility, in addition to more robust epidemiological information on common causes of infection in the region of practice, additional diagnostic capacity to positively identify more diagnoses, and attention to broader systemic and contextual factors [17–19].

In addition, the intervention-arm diagnostic and treatment algorithm allowed for clinical judgment on the part of the treating clinician rather than strict adherence to the results of diagnostic tests. This is important as the nuances of individual cases cannot adequately be addressed in a simple algorithm, but again, experience implementing malaria RDTs in similar settings has shown a range of adherence to test results in prescribing practices [20, 21]. Adherence to new diagnostic strategies may change with further implementation work, changes in test availability and costs (although costs were not assessed in this study), and clinician and patient familiarity over time.

Finally, the impact of the intervention on antibiotic prescribing in our study may have been limited by the study design. While many patients were enrolled, just a few clinicians were responsible for diagnosis and prescribing decisions—1 clinician per site for the intervention arm and 2 or 3 clinicians per site for the control arm. These staffing levels were appropriate for the typical patient flow at each health center but mean that individual clinician preferences could have had an outsized influence on diagnoses made and prescribing practices observed. Furthermore, the intervention- and control-arm activities at each site were conducted in the same building; however, the clinical and laboratory staff for each arm were different personnel who worked in separate rooms, reducing the risk of cross-contamination. In addition, our results may not be directly extrapolatable to the potential uptake of the POCT panel and strategy by clinicians working under routine conditions at Ugandan health centers, because intervention-arm clinicians were hired specifically for the study. This was necessary to avoid overloading the government-employed staff who provide routine care at participating health centers, but the issue of how to sustainably incorporate diagnostics into routine care in these settings needs to be addressed in any future implementation work.

The second main objective of this study was to evaluate the impact of the intervention on patients' clinical outcomes. The study was conducted only among outpatients who presented without signs of severe illness, a group for whom, in general, poor outcomes are rare, and fortunately, the large majority of patients at all sites and study arms had favorable outcomes. The fact that statistically significant differences were not seen between study arms, even within subgroup analyses stratified by age, presenting syndrome, and other features, may be explained by the fact that many outpatient febrile illnesses are likely due to self-limiting causes—viral, bacterial, or other—that eventually would resolve satisfactorily without specific treatment [22, 23].

A strength of this study is its inclusion of both adult and child patients older than 1 year, allowing the assessment of potential prescribing and outcome differences across age groups. In keeping with observations elsewhere [24, 25], in this study antibiotics were prescribed commonly for febrile outpatients of all ages, while age-related prescribing differences between study arms were not consistent across the 3 sites. Similarly, there were no age-related differences between study arms in clinical outcomes. Another strength of the study is that it was conducted in sites of known and varied malaria transmission intensity [10–12]. Malaria is an important cause of healthcare-seeking and morbidity, and a driver of antimicrobial medicine use, in Uganda [26]. In most regions of the country, including our 3 study sites, transmission is holoendemic and relatively high by global standards; indeed, between 28% and 54% of all enrolled patients tested positive by mRDT. Somewhat surprisingly, the strongest and most consistent prescribing differences between study arms were correlated with malaria status: at all 3 sites, malaria-positive patients in the intervention arm were significantly more likely to receive an antibiotic prescription than malaria-positive patients in the control arm. In Burkina Faso and Ghana, the 2 other African sites that participated in this multicenter study (reported elsewhere in this supplement issue), a similar trend was observed of significantly less antibiotic prescribing for malaria-negative patients. However, to our knowledge, this finding has not been reported in other settings previously. Indeed, most studies to date have shown a tendency towards more empiric antibiotic prescription for febrile patients who test negative for malaria [14, 27–29]. We hypothesize that the clinicians in the intervention arm may have been uncomfortable withholding an antibiotic—as guided by a strict interpretation of the algorithm (Figure 1, Tables 4 and 5)—in the face of an elevated CRP (and/or CBC) result (Table 6), which may be seen in malaria-positive patients, leading to higher-than-usual antibiotic prescribing in this subgroup. This observation should be investigated further and addressed in future implementation work.

More amoxicillin was prescribed for intervention-arm patients, presumably due to algorithm guidance for respiratory syndromes. Antibiotic choice for empiric prescribing varied across sites, likely due to local availability, individual health facility and clinician preferences, as was seen in control arms in this study and in other studies in similar settings [14, 30].

In conclusion, this study found that a diagnostic intervention for the management of febrile outpatients did not achieve the desired impact on antibiotic prescribing at 3 diverse and representative sites in Uganda. Further work is needed to design and evaluate effective strategies to safely reduce inappropriate antibiotic use and improve antibiotic targeting for patients with acute febrile illnesses in similar settings.

## Data Availability

Data are available from the corresponding author on request.

## Notes

***Author Contributions*.** J. K., A. S., F. E. K., D. K.-M., P. H., O. S., S. D., C. E. M., P. O., E. R., P. A., and H. H. were involved in the conception and design of the study. J. K., A. S., D. K.-M., D. E.-S., F. E. K., E. R., P. A., and H. H. were involved in data acquisition. S. W., T. K., and J. N. were involved in data analysis. J. K. and H. H. wrote the first draft of the manuscript. All authors were involved in data interpretation and refining the manuscript, and all authors reviewed and approved the final version of the manuscript.

***Acknowledgments*.** The authors are grateful to participating patients and caregivers and to staff of the health centers for their generous collaboration; to the leadership of the districts where the study sites are located; to the national AMR subcommittee; to the study staff and to the administrative staff of the Infectious Diseases Research Collaboration (IDRC) in Uganda; to Ana Belen Ibarz and Cecilia Ferreyra for helpful comments on manuscript drafts; and to Stuart Wakelin for medical writing assistance.

***Financial support*.** This work was supported by dedicated UK Foreign, Commonwealth and Development Office and the Swiss Agency for Development and Cooperation.

***Supplement sponsorship*.** This article appears as part of the supplement “Using Diagnostic Tools to Support Antimicrobial Stewardship and Improve Outcome in Resource-Limited Contexts,” sponsored by FIND.

## References

[1] World Health Organization. Global Action Plan on Antimicrobial Resistance. Available at: https://www.who.int/publications-detail-redirect/9789241509763. Accessed 4 December 2022.

[2] World Health Organization. Uganda: Antimicrobial Resistance National Action Plan 2018–2023. Available at: https://www.who.int/publications/m/item/uganda-antimicrobial-resistance-national-action-plan-2018-2023. Accessed 4 December 2022.

[3] Antimicrobial Resistance Collaborators . Global burden of bacterial antimicrobial resistance in 2019: a systematic analysis. Lancet 2022; 399:629–55. 35065702 10.1016/S0140-6736(21)02724-0 PMC8841637

[4] GARP—Uganda Situation Analysis . Available at: https://onehealthtrust.org/publications/reports/garp-uganda-situation-analysis/. Accessed 4 December 2022.

[5] Mugerwa I, Nabadda SN, Midega J, Guma C, Kalyesubula S, Muwonge A. Antimicrobial resistance situational analysis 2019–2020: design and performance for human health surveillance in Uganda. Trop Med Infect Dis 2021; 6:178. 34698282 10.3390/tropicalmed6040178 PMC8544686

[6] Okello N, Oloro J, Kyakwera C, Kumbakumba E, Obua C. Antibiotic prescription practices among prescribers for children under five at public health centers III and IV in Mbarara district. PLoS One 2020; 15:e0243868. 10.1371/journal.pone.0243868 PMC7769467 33370280

[7] Dhesi Z, Enne VI, O’Grady J, Gant V, Livermore DM. Rapid and point-of-care testing in respiratory tract infections: an antibiotic guardian? ACS Pharmacol Transl Sci 2020; 3:401–17. 32551433 10.1021/acsptsci.0c00027 PMC7233852

[8] Visser T, Daily J, Hotte N, Dolkart C, Cunningham J, Yadav P. Rapid diagnostic tests for malaria. Bull World Health Organ 2015; 93:862–6. 26668438 10.2471/BLT.14.151167 PMC4669726

[9] Staveteig S, Wang S, Head SK, Bradley SEK, Nybro E. Demographic patterns of HIV testing uptake in sub-Saharan Africa. 2013. Available at: https://dhsprogram.com/publications/publication-cr30-comparative-reports.cfm. Accessed 4 December 2022.

[10] Raouf S, Mpimbaza A, Kigozi R, et al Resurgence of malaria following discontinuation of indoor residual spraying of insecticide in an area of Uganda with previously high-transmission intensity. Clin Infect Dis 2017; 65:453–60. 28369387 10.1093/cid/cix251 PMC5850037

[11] Epstein A, Namuganga JF, Kamya EV, et al Estimating malaria incidence from routine health facility-based surveillance data in Uganda. Malar J 2020; 19:445. 33267886 10.1186/s12936-020-03514-z PMC7709253

[12] Sserwanga A, Harris JC, Kigozi R, et al Improved malaria case management through the implementation of a health facility-based sentinel site surveillance system in Uganda. PLoS One 2011; 6:e16316. 10.1371/journal.pone.0016316 PMC3023768 21283815

[13] Salami O, Horgan P, Moore CE, et al Impact of a package of diagnostic tools, clinical algorithm, and training and communication on outpatient acute fever case management in low- and middle-income countries: protocol for a randomized controlled trial. Trials 2020; 21:974. 33239106 10.1186/s13063-020-04897-9 PMC7687811

[14] Hopkins H, Bruxvoort KJ, Cairns ME, et al Impact of introduction of rapid diagnostic tests for malaria on antibiotic prescribing: analysis of observational and randomised studies in public and private healthcare settings. BMJ 2017; 356:j1054. 10.1136/bmj.j1054 PMC5370398 28356302

[15] Bruxvoort KJ, Leurent B, Chandler CIR, et al The impact of introducing malaria rapid diagnostic tests on fever case management: a synthesis of ten studies from the ACT consortium. Am J Trop Med Hyg 2017; 97:1170–9. 28820705 10.4269/ajtmh.16-0955 PMC5637593

[16] Burchett HED, Leurent B, Baiden F, et al Improving prescribing practices with rapid diagnostic tests (RDTs): synthesis of 10 studies to explore reasons for variation in malaria RDT uptake and adherence. BMJ Open 2017; 7:e012973. 10.1136/bmjopen-2016-012973 PMC5353269 28274962

[17] Cox JA, Vlieghe E, Mendelson M, et al Antibiotic stewardship in low- and middle-income countries: the same but different? Clin Microbiol Infect 2017; 23:812–8. 28712667 10.1016/j.cmi.2017.07.010

[18] Hopkins H, Bassat Q, Chandler CI, et al Febrile Illness Evaluation in a Broad Range of Endemicities (FIEBRE): protocol for a multisite prospective observational study of the causes of fever in Africa and Asia. BMJ Open 2020; 10:e035632. 10.1136/bmjopen-2019-035632 PMC7375419 32699131

[19] Basu S, Copana R, Morales RJ, et al Keeping it real: antibiotic use problems and stewardship solutions in low- and middle-income countries. Pediatr Infect Dis J 2022; 41:S18. 35134036 10.1097/INF.0000000000003321 PMC8815843

[20] O’Boyle S, Bruxvoort KJ, Ansah EK, et al Patients with positive malaria tests not given artemisinin-based combination therapies: a research synthesis describing under-prescription of antimalarial medicines in Africa. BMC Med 2020; 18:17. 31996199 10.1186/s12916-019-1483-6 PMC6990477

[21] Madut DB, Rubach MP, Bonnewell JP, et al Trends in fever case management for febrile inpatients in a low malaria incidence setting of Tanzania. Trop Med Int Health 2021; 26:1668–76. 34598312 10.1111/tmi.13683 PMC8639662

[22] Tam P-YI, Obaro SK, Storch G. Challenges in the etiology and diagnosis of acute febrile illness in children in low- and middle-income countries. J Pediatr Infect Dis Soc 2016; 5:190–205. 10.1093/jpids/piw016 PMC7107506 27059657

[23] Maze MJ, Bassat Q, Feasey NA, Mandomando I, Musicha P, Crump JA. The epidemiology of febrile illness in sub-Saharan Africa: implications for diagnosis and management. Clin Microbiol Infect 2018; 24:808–14. 29454844 10.1016/j.cmi.2018.02.011 PMC6057815

[24] Obakiro SB, Napyo A, Wilberforce MJ, et al Are antibiotic prescription practices in eastern Uganda concordant with the national standard treatment guidelines? A cross-sectional retrospective study. J Glob Antimicrob Resist 2022; 29:513–9. 34890831 10.1016/j.jgar.2021.11.006

[25] Allwell-Brown G, Hussain-Alkhateeb L, Kitutu FE, Strömdahl S, Mårtensson A, Johansson EW. Trends in reported antibiotic use among children under 5 years of age with fever, diarrhoea, or cough with fast or difficult breathing across low-income and middle-income countries in 2005–17: a systematic analysis of 132 national surveys from 73 countries. Lancet Glob Health 2020; 8:e799–807. 32446345 10.1016/S2214-109X(20)30079-6

[26] Yeka A, Gasasira A, Mpimbaza A, et al Malaria in Uganda: challenges to control on the long road to elimination: I. Epidemiology and current control efforts. Acta Trop 2012; 121:184–95. 21420377 10.1016/j.actatropica.2011.03.004 PMC3156969

[27] Mubi M, Kakoko D, Ngasala B, et al Malaria diagnosis and treatment practices following introduction of rapid diagnostic tests in Kibaha District, coast region, Tanzania. Malar J 2013; 12:293. 23977904 10.1186/1475-2875-12-293 PMC3765530

[28] Ndhlovu M, Nkhama E, Miller JM, Hamer DH. Antibiotic prescribing practices for patients with fever in the transition from presumptive treatment of malaria to ‘confirm and treat’ in Zambia: a cross-sectional study. Trop Med Int Health 2015; 20:1696–706. 26311240 10.1111/tmi.12591

[29] Johansson EW, Selling KE, Nsona H, et al Integrated paediatric fever management and antibiotic over-treatment in Malawi health facilities: data mining a national facility census. Malar J 2016; 15:396. 27488343 10.1186/s12936-016-1439-7 PMC4972956

[30] Allwell-Brown G, Namugambe JS, Ssanyu JN, et al Patterns and contextual determinants of antibiotic prescribing for febrile under-five outpatients at primary and secondary healthcare facilities in Bugisu, eastern Uganda. JAC Antimicrob Resist 2022; 4:dlac091. 10.1093/jacamr/dlac091 PMC9444054 36072304

