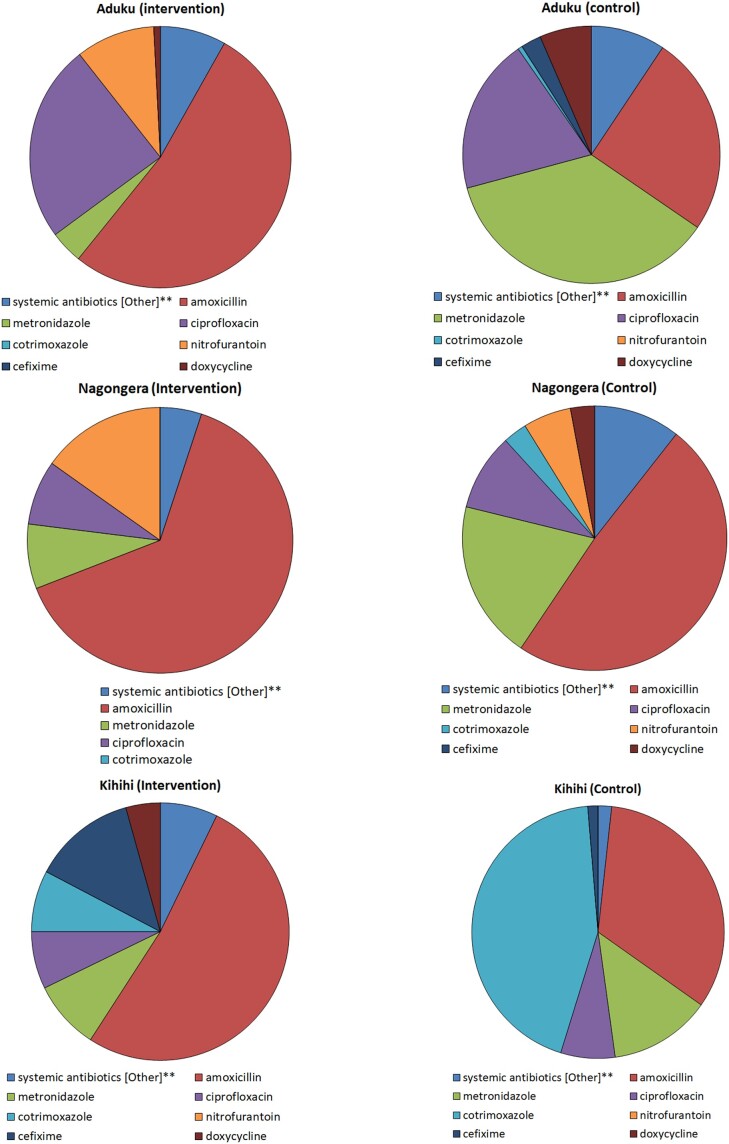# Correction to: Impact of the Introduction of a Package of Diagnostic Tools, Diagnostic Algorithm, and Training and Communication on Outpatient Acute Fever Case Management at 3 Diverse Sites in Uganda: Results of a Randomized Controlled Trial

**DOI:** 10.1093/cid/ciad733

**Published:** 2024-02-26

An error appeared in the supplement published with the 15 July 2023 issue of the journal (Kapisi et al. “Impact of the introduction of a package of Diagnostic tools, Diagnostic algorithm, and Training and Communication on Outpatient Acute Fever case management at 3 diverse sites in Uganda: Results of a Randomized Controlled Trial.” *Clin Infect Dis*; 2023 Jul 15; 77(Suppl 2): S156–S170. Published online 2023 Jul 25. doi: 10.1093/cid/ciad341). In Figure 4, the first 2 pie charts represent Aduku Health Centre IV. The pie chart for the Intervention arm should be on the left side, and the one for the control arm should be on the right. Also, the second set of pie charts has an error in the name of the health centres. On the left, it should be Nagongera (intervention) and on the right, it should be Nagongera (control). The corrected figure file appears below.

**Figure:**